# Bacteriophages and Their Immunological Applications against Infectious Threats

**DOI:** 10.1155/2017/3780697

**Published:** 2017-04-16

**Authors:** Elena Criscuolo, Sara Spadini, Jacopo Lamanna, Mattia Ferro, Roberto Burioni

**Affiliations:** ^1^Microbiology and Virology Unit, Vita-Salute San Raffaele University, Milan, Italy; ^2^Vita-Salute San Raffaele University, Milan, Italy; ^3^Neurobiology of Learning Unit, Division of Neuroscience, San Raffaele Scientific Institute, Milan, Italy; ^4^Laboratory of Microbiology and Virology, San Raffaele Hospital, Milan, Italy

## Abstract

Bacteriophage therapy dates back almost a century, but the discovery of antibiotics led to a rapid decline in the interests and investments within this field of research. Recently, the novel threat of multidrug-resistant bacteria highlighted the alarming drop in research and development of new antibiotics: 16 molecules were discovered during 1983–87, 10 new therapeutics during the nineties, and only 5 between 2003 and 2007. Phages are therefore being reconsidered as alternative therapeutics. Phage display technique has proved to be extremely promising for the identification of effective antibodies directed against pathogens, as well as for vaccine development. At the same time, conventional phage therapy uses lytic bacteriophages for treatment of infections and recent clinical trials have shown great potential. Moreover, several other approaches have been developed in vitro and in vivo using phage-derived proteins as antibacterial agents. Finally, their use has also been widely considered for public health surveillance, as biosensor phages can be used to detect food and water contaminations and prevent bacterial epidemics. These novel approaches strongly promote the idea that phages and their proteins can be exploited as an effective weapon in the near future, especially in a world which is on the brink of a “postantibiotic era.”

## 1. Introduction

Bacteriophages (or phages), small viruses of about 20–200 nm in size, are probably the most ancient and ubiquitous existing organisms on Earth. They date back 3 billion years, and they specifically infect bacteria to replicate, therefore playing an important role in maintaining the equilibrium of every ecosystem where bacteria exist [1].

Despite controversy over claims for priority, bacteriophage discovery is independently attributed to both F.W. Twort (1915) and to F. H. d'Herelle (1917) [2–4]. The former observed a peculiar in vitro transformation of micrococcus colonies, “plaques or rings grew and the disease could be transferred through simple contact between colonies.” In 1917, at Pasteur Institute in Paris, researcher d'Herelle discovered an infective agent able to selectively destroy cultures of *Shigella dysenteriae* bacteria. The microorganism responsible for that lysis was called “bacteriophage,” coined by the combination of *bacteria* and the Greek *phagein*, which means *devour* [5].

Phage classification is based on morphology, nucleic acid characteristics, and properties, although other factors such as host spectrum, virus-host interaction, and immunological features should be considered [6]. Concerning morphology, there are phages with icosahedral proteic capsid and no tail, phages with contractile tails, phages without contractile tails, and filamentous phages. The specificity for the *E. coli* conjugative F pilus was found to be a distinguishing trait of a large group of phages (Ff), including the first isolated filamentous phages f1 [7–10]; many other species, including temperate and Gram-positive tropic ones, were later discovered [11].

Lytic phages, unlike temperate and filamentous phages, lyse the bacterial cell at the end of their life cycle, disrupting its metabolism and releasing newly formed phage particles. During the lytic cycle, an ecliptic phase occurs: the bacterial cell does not contain any complete virion yet, but the virus replicates and both host and early viral components are involved. Late viral proteins are instead structural elements necessary for new particle assembly and formation and for the lysis of the cell that occurs after maturation [12].

During the lysogenic cycle, the viral genome does not take control of host machinery but remains inside the cell and replicates together with the host genome in order to generate clones of infected cells, which are then able to grow and divide for long periods of time. Latent forms of the viral genome are named “prophage” [12].

Phage discovery occurred well before the development of antibiotics, thus raising interest within the worldwide scientific community.

By 1919, d'Herelle and his colleagues in Paris began using bacteriophages in a therapeutic way, launching the “phage therapy” [13, 14]. That first, small-scale clinical trial concerned four children successfully treated from bacterial dysentery; after receiving a single dose of phage preparation, all began to recover within 24 hours. At the same time, phage sample ingestion by healthy individuals proved the treatment safety. In 1921, Bruynoghe and Maisin published the first paper describing the efficacy of bacteriophages in the treatment of a staphylococcal skin infection: they injected the phage preparation around surgically opened lesions and the infection regressed within 24–48 hours [15].

Bacteriophage therapy rapidly developed globally and attracted the attention of pharmaceutical companies and, independently, the Russian and German army physicians, who started using phages to treat soldiers.

Nevertheless, those results were sometimes controversial, and antibiotics were discovered and industrially produced [13, 14]. Thus, enthusiasm for phage therapy began to decrease in the West during the 1940s and the 1950s, even if in the meanwhile Luria and Delbruck used bacteriophages as model organisms for their “Fluctuation test,” leading to the understanding of the genetic basis of interactions between viruses and hosts and to the development of the first molecular techniques [16, 17]. However, scientific interest for bacteriophages arose again in the late 1980s, with the design of a novel technique: phage display. Since then, this application has proved to be extremely successful for the identification of monoclonal antibodies (mAbs) directed against a wide range of pathogens, and several companies currently use this technique for the discovery of their lead compounds.

A recent report from 2014, commissioned by the UK government, predicts the killing of 10 million people across the world by 2050 because of antimicrobial-resistant infections; the Centers for Disease Control and Prevention (CDC) categorized some bacteria as presenting urgent, serious, and concerning threats [18]. Therefore, the appearance of multidrug-resistant pathogens has renewed the importance of pioneering antibacterial strategies in order to prevent epidemic infections, with a close monitoring of hospitals as well as food industries. This is giving a boost in revisiting the practical applications of bacteriophages both for phage therapy and for rapid and precise detection of bacterial pathogens.

## 2. Phage Display Technique for mAbs Identification against Pathogens

Phage display technique was invented in 1985: since then, it has been successfully applied to many immunology domains, in particular against infectious diseases and cancer research [6]. This technique has led to important results in both translational and basic research, with the identification of mAbs and small peptides capable of inhibiting functions of their receptor target. Some of these molecules have been developed as therapeutics and are currently in clinical or preclinical trials; on the other hand, others have been crucial to describe protein-protein interactions and revealed important therapeutic targets [19, 20].

Briefly, this method focuses on the construction of a library of peptides or antibody variants, which are then selected for their affinity to the target of interest since they are fused to a phage-coat protein. All surface proteins of bacteriophages can be engineered for display, but the most used are pVIII and pIII from M13 filamentous phages. In fact, each virion contains about 2700 copies of the former protein, representing almost 87% of its mass, and they are half-exposed to the environment, thus allowing an efficient display of only short-sequence peptides due to virion architecture. On the other hand, pIII can be used for larger peptides, resulting only in a slight loss of infectivity in a few cases [21]. Each library is composed by phagemid vectors containing only the sequence of a phage-coat protein fused to the peptide of interest; therefore, a helper phage with a reduced packaging efficiency is needed in order to obtain a population of phages both infectious and composed by modified coating proteins. The biopanning procedure is then performed and phages are selected for their ability to bind the antigen of interest. Many factors must be taken into account: library variability, target conformation, affinity, and avidity of the molecules exposed on phages. As mentioned, phage display has been widely used to find novel therapeutics against pathogens, particularly mAbs. This has been possible through two different biopanning strategies: using specific molecular targets, such as enzymes or membrane receptors, or using whole viruses or bacterial cells. The main difference between these two approaches is that the second one allows the identification of pathogen structures not already identified as drug targets. Moreover, surface antigens often present motifs able to elicit non-neutralizing mAbs and elude host immune response, so the screening procedure of the biopanning outcome must be done properly in order to identify only the few effective molecules.

Several human infectious diseases have been successfully addressed for drug discovery using phage display. Using molecular targets, specific mAbs have been isolated against viruses such as HCV, HIV, HBV, JCV, HSV, influenza A, or bacteria like *H. pylori* [20, 22–29], while whole-cell approach resulted in the identifications of effective molecules against rabies virus, *L. monocytogenes*, *H. pylori*, *C. trachomatis*, and various *Bacillus* species [30–34].

As stated above, many of the molecules developed using phage display technique are currently in clinical trials or under evaluation at a preclinical stage. MedImmune developed motavizumab, a version of their lead compound against RSV infection (palivizumab) which was optimized by enhancing its affinity to the target [35]. Affitech A/S is a company whose phagemidic library has a diversity of 10^10^, allowing the identification of several human mAbs, for example, bavituximab has been tested in several clinical trials against HCV and HIV chronic infections, and for the treatment of several types of cancer [36]. Its target is phosphatidylserine, a molecule that is located in the inner part of the cell membrane of healthy cells, but becomes exposed on the surface of infected cells or solid cancer cells, allowing their immune evasion. Moreover, Human Genome Sciences developed raxibacumab for anthrax using a part of the toxin itself, the *B. anthracis*-recombinant protective antigen protein [37]. Additionally, Isogenica developed an improved version of the phage display technique, called “CIS display”: it provides DNA fragments encoding the peptides of the library fused to the gene of RepA, the DNA replication initiator protein, so that proteins or peptides are displayed in vitro directly bound to their encoding DNA [38].

## 3. Phages for Vaccine Development

Neutralizing mAbs are very useful as therapeutics but can also be of great importance for the identification of protective epitopes on pathogen structures. In fact, the characterization of these regions on viruses and bacteria could suggest which elements should be included into a vaccine formulation in order to be effective. To date, there are several human infectious diseases that cannot be treated with vaccination, since all the approaches tested so far proved to be unsuccessful. One of the causes of failure is the nature of the antigens included into the vaccine. In fact, the use of recombinant proteins may limit, though not eliminate, the mechanisms of immunodominance that pathogens use to evade host immune response. Immunodominant epitopes are those regions that can be mutated without affecting the pathogen protein functions, both rerouting the adaptive immune response against non-neutralizing epitopes and masking epitopes that can impair infection mechanisms. Thus, bacterial and viral proteins often elicit a varying humoral response, with a minimal neutralizing fraction, unable to defeat the infection [39–41]. Moreover, also antibody-mediated interference has been widely described [22, 42]. Dulbecco et al. first hypothesized the interfering effect of non-neutralizing Abs (non-nAbs) to explain the apparent inhibition of virus neutralization exerted by some serum samples [43]. Later observations in both chronic and acute infections confirmed this assumption. Two mechanisms have been proposed for this evasion strategy: non-nAbs interference by steric hindrance due to proximity of neutralizing and non-neutralizing epitopes; inhibition of binding of nAbs due to epitope conformational changes caused by non-nAbs binding to the pathogen protein. Furthermore, it has been speculated that non-nAbs may enhance infections through interaction with Fc receptors or complement receptors [44].

On the other hand, neutralizing mAbs as molecular probes could be extremely useful for a rational design of vaccine formulations: the administration of their mimotopes should elicit only an effective humoral response against pathogens [45, 46]. For this purpose, phage display technique has been widely employed: HCV, HIV, and *Plasmodium falciparum* proteins are just examples of molecular targets used in the biopanning procedure for epitope mapping [26, 47, 48].

In addition, phage particles themselves can induce both cellular and humoral immune response when recognised by the immune system [49]. Moreover, they proved to be unaffected by harsh physical and chemical conditions, resulting suitable for vaccine delivery [50]. In fact, these peculiar characteristics, as well as cost-effective production and ease of modification, made bacteriophages attractive for industrial development of phage-based vaccines. Two different approaches have been developed: the first one is based on phage particles with antigens transcriptionally fused to their coat proteins, resembling the phage display technique described above. This strategy proved to be effective against *Yersinia pestis* [51], where T4 phages displayed an engineered structural subunit of the pathogen on its capsid, and against HIV and influenza, using phage T4 and T7, respectively [52, 53]. The second approach consists of phage DNA vaccines: the antigen gene is delivered by phages into antigen-presenting cells to be expressed and processed by them, leading to enhanced immune response compared to naked DNA delivery. Vaccines against HBV and HSV type 1 infections have been developed using an engineered phage *λ* that carries a viral surface epitope gene [49, 54]. More recently, a phage *λ*-based vaccine against *Chlamydia abortis* elicited an immune response superior to the stimulation of the commercial vaccine Enzovax [55].

## 4. Phage Therapy: Last Updates

Due to their high level of organization, their unique morphology, and biological properties, bacteriophages appear as sophisticated nanomachines and, as described above, have been immediately employed for therapeutic purposes.

Typically, only lytic phages are exploited for phage therapy: firstly, because they kill the host bacteria in a more efficient manner; subsequently because, after lysogenic induction, temperate phages can transfer bacteria DNA fragments to other species, and if these fragments contain gene-encoding toxins or antibiotic resistance elements, they could generate new dangerous bacteria. On the contrary, lytic phages are unable to perform transduction, thus ensuring a safer profile.

Nowadays, many bacteria have collected multiple resistance mechanisms, thus rendering some antibiotic classes useless. Beyond the quite high level of natural resistance, patients are concomitantly treated with broad-spectrum antibiotic molecules, increasing the rate of blind selection of multidrug resistance isolates (MDR). In hospital settings, *Enterococcus faecium*, *Staphylococcus aureus*, *Klebsiella pneumoniae*, *Acinetobacter baumannii*, *Pseudomonas aeruginosa*, and *Enterobacter cloacae* (ESKAPE) are examples of multidrug-resistant strains that solicit novel therapeutic measures [56]. This challenge forced modern medicine to review methods against bacterial infections, reconsidering the beneficial potential of phages.

In 2006, the Food and Drug Administration (FDA) approved the first Phase I clinical trial evaluating the safety of an eight-phage cocktail able to kill *Staphylococcus aureus*, *Pseudomonas aeruginosa*, *and Escherichia coli*; 42 patients with venous leg ulcers were treated and a high-safety profile was demonstrated (ClinicalTrials.gov Identifier: NCT00663091) (Table 1).

In 2009, another study assessed safety, tolerability, and efficacy of oral administration of T4 phages in young children with diarrhea due to enterotoxigenic *Escherichia coli* (ETEC) and/or enteropathogenic *Escherichia coli* (EPEC) infections. This study aimed at demonstrating the potential of a novel therapy for childhood diarrhea, a major cause of morbidity and deaths in Bangladesh and other developing countries, and thus a priority for improving child health: as shown in Table 1, oral phages showed a safe gut transit, even if it failed to achieve intestinal amplification and did not improve diarrhea outcome. This was possibly related to insufficient phage coverage and too low *E. coli* titres requiring higher oral phage dosage (ClinicalTrials.gov Identifier: NCT00937274).

Afterwards, a randomized, multicentric, open-label Phase I/II clinical trial started in 2015 is currently in a “recruiting phase.” The aim of this trial is to assess tolerance and efficacy of a local bacteriophage treatment of wound infections due to *Escherichia coli* or *Pseudomonas aeruginosa* in burn patients. The treatment uses GMP-produced cocktails of anti-*E. coli* and anti-*P. aeruginosa* bacteriophages (Pherecydes Pharma), and the safety profile of this phage therapy will be compared to the safety profile of a standard of care. This is a European Research & Development (R&D) project funded by the European Commission under the 7th Framework Programme for Research and Development involving seven clinical sites in the EU (ClinicalTrials.gov Identifier: NCT02116010).

Finally, a French trial which is scheduled to start in 2017 aims to compare the efficacies of a standard treatment with the addition of phage therapy versus a standard treatment plus placebo for diabetic foot ulcers monoinfected by *Staphylococcus aureus*. It will be a randomized, multicentric, controlled, two-parallel-group, double-blind, superiority trial and will enrol 60 subjects (ClinicalTrials.gov Identifier: NCT02664740).

Phage therapy efficacy is still controversial and is practiced only in a few institutions worldwide, with the Eliava Institute of Bacteriophage (Tbilisi, Georgia) and the Institute of Immunology and Experimental Therapy of Polish Academy of Sciences (Wroclaw, Poland) being the leading centers; nevertheless, as mentioned above, many pharmaceutical companies involved in phage research are carrying out an increasing number of clinical trials.

As reviewed by Oliveira and colleagues, despite a growing appreciation for bacteriophages, different human bacterial pathogens remain to be targeted by phage preparations [59]. Among these, *Rickettsia*, *Ehrlichia*, and *Coxiella*, which can potentially cause severe diseases, should be investigated with the purpose of developing a competent phage therapy.

Neither immunological complications nor adverse effects have been shown after administration of extensive amounts of bacteriophages, but their usage in vivo can give rise to immune responses. For example, innate immunity and phagocytosis in the blood and liver can reduce phage titres after intravenous administration. An enhancement of major classes of immunoglobulin can also be induced, due by long-term treatments or phage exposure itself, and this can decrease the availability of active phages, reducing the effectiveness of the therapeutic approach. In order to prevent these undesirable immunological effects in the medical use, every individual phage immune response should be assessed, paying specific attention to route of administration, dose, accompanying compounds, and timing of exposure [60, 61].

At present, the choice of phage formulation is under debate because of the lack of information concerning the therapeutic outcome under different conditions. A simple aqueous formulation is the most applied, but long-term stability studies of different phage formulations would be pivotal to ensure the endurance of their therapeutic effect. Furthermore, an accurate study addressing the pharmacokinetic profile of phage preparations is essential for clinical applications. Indeed, phages are thought to control bacterial infections by means of “active” and “passive” treatments: the former one involves a secondary phage infection of bacteria before they are killed, while for the latter one, the initial dose of phages is sufficient.

Concerning phage therapy delivery, the parenteral (and more specifically intraperitoneal) route seems to be the most successful form of systemic administration; oral delivery was proved to be preferable for gastrointestinal *E. coli* infections, although the highly acidic and proteolytically active environment of the stomach represents a major issue since it undermines phage stability.

Moreover, timing of administration and concentration of phages are critical aspects affecting clinical results, and thus, they should be rigorously optimized by dedicated research for each phage or cocktail of phages. In this context, phage stability within different formulations should be taken into account to avoid any unacceptable loss of activity during the treatment [62].

## 5. Phage-Derived Proteins as Antibacterial Agents

The huge progress that has been made in molecular biology has opened up new possibilities in the design of recombinant phage-derived proteins. Encouraging results emerged for phage enzymes involved in the first step of viral infection responsible for bacterial envelope degradation, named depolymerases. Furthermore, proteins encoded by lysis-cassette genes (as holins and endolysins), which are responsible for progeny release during the phage lytic cycle, are arousing growing interest too. The main characteristics of these antibacterial agents and the results obtained regarding their biological activity in vitro and in vivo will be addressed in the next sections.

### 5.1. Polysaccharide Depolymerases

As mentioned above, polysaccharide hydrolases (depolymerases) are enzymes used by phages for bacterial cell degradation, in particular targeting macromolecule carbohydrates within extracellular polysaccharides (EPS) and lipopolysaccharides (LPS) [63]. Polysaccharides are of particular importance for survival of many encapsulated bacteria, as they promote host virulence by letting cell adherence to both biotic and abiotic surfaces, or by protecting cells from phagocytosis and antimicrobials [64]. They also represent a physical barrier to phages, protecting the receptors needed for adsorption and infection. Depolymerases are the enzymes they use for “stripping away” the extracellular bacterial polysaccharide, and they have therefore been tested as therapeutics [65]. To date, few therapeutic protocols have been tried in vivo. Dubos and Avery in 1931 were the first to demonstrate that these enzymes could protect mice from type III pneumococcal infection, acting on the capsule polysaccharides [66]. Later, other protection studies in mice, rabbits [67], and monkeys [68] described the control of lethal pneumococcal infections by a partially purified preparation of depolymerases. Finally, Mushtaq et al. showed that intraperitoneal administration of 0.25 mg (≫0.83 mg/kg) of the endosialidase E-protected rats from neurotropic strains of *E. coli* infection [69], and Scorpio et al. similarly found that depolymerase capsule removal from *Bacillus anthracis* resulted in reduced resistance to phagocytosis and associated killing [70], protecting a mouse infection model [71].

Furthermore, these phage-derived enzymes may have a greater potential as therapeutic agents against biofilm formation, as the structural backbone of glycocalix is composed of bacterial EPS [72, 73]. Depolymerases can be effective in EPS removal, making the biofilm bacteria more susceptible to treatment by antimicrobials.  Table 2 shows a list of studies where bacteriophages and their EPS depolymerases have been employed to fight experimental bacterial biofilms. All studies involved in vitro growth of biofilms.

### 5.2. Endolysins

Endolysins are enzymes produced at the end of the lytic phage lifecycle: they accumulate in the cytoplasm of the host bacterium until they translocate through holes formed by holins in the plasma membrane. Then, endolysins cleave peptidoglycan bonds in the cell wall causing cell bursting and release of progeny phages [81]. Their biological activity is of particular interest as they are able to lyse a target cell within seconds after contact [82, 83], causing holes in the bacterial wall with its consequent osmotic lysis and death [84]. These proteins are among the most active and safest substances able to kill bacteria, but their activity has a major constraint. In fact, endolysin antibacterial activity is particularly effective against Gram-positive bacteria, since they lack an outer membrane, unlike Gram negatives [85].

Lysin structure reflects this dissimilar biological activity. Endolysins derived from Gram-positive-infecting phages have a modular domain structure that shows a variety in its architecture [86]. It is composed of two highly conservative domains: a N-terminal catalytic domain and a C-terminal bacterial wall-binding domain, connected by a linker [87]. Endolysins can be divided into five different classes according to their enzymatic activity: (1) N-acetylmuramidases (lysozymes), (2) endo-*β*-N-acetylglucosaminidases, (3) lytic trans-glycosylases, (4) endopeptidases, and (5) N-acetylmuramyl-L-ala-amidases. Classes 1 to 3 can cleave sugars and class 4 peptides and class 5 serve to cleave peptide bonds between sugars and peptides. All endolysins are hydrolases, except for transglycolases; amidases and muramidases are the most represented classes [88]. The C-terminal domain specifically binds ligands on the bacterial wall, tethering the lysin to the proteoglycan: even if the number of binding domains varies between endolysins [86], the affinity is almost as strong as antigen-antibody binding [89].

On the contrary, lysins from phages that infect Gram-negative bacteria mainly present a globular structure and lack of cell wall-binding domain [90].

The modular structure of endolysins suggested the opportunity to engineer novel enzymes in order to improve their bacteriolytic potency, to broaden their lytic spectrum, or to avoid phage resistance. For example, chimeric enzymes (chimeolysins) can be obtained by substitution or addition of heterologous binding domains to expand the bacteriolytic spectrum outside the native endolysins species specificity [86, 91]. Further improvements to endolysin properties have been obtained so far after fusion to a peptide or a protein domain of nonendolysin origin (artilysins) [92]. Recent studies led to the design of some effective hybrid enzymes targeting Gram-negative pathogens: for example, a chimera of T4 lysozyme fused to the bacterial toxin pesticin, targeting FyuA, a major virulence factor for some *Yersinia* and pathogenic *E. coli* strains [93]; another artilysin was designed against multidrug-resistant strains and persisters of *Pseudomonas aeruginosa* by fusing to the N-terminus of an endolysin, a sheep myeloid antimicrobial peptide, able to pass the outer membrane of Gram-negative bacteria via a self-promoted uptake pathway [94]. Finally, modular endolysins from Gram-negative-infecting phages are rather rare but endowed with stronger and faster activity than the globular ones [95]. They might represent potential candidates to control multidrug-resistant infections, and domain swapping may allow creating novel enzymes with higher specificity or efficiency, as for chimeolysins obtained from Gram-positive-infecting phages.

Purified endolysin activity against bacterial was first described in 1959 [96]. Since then, several endolysins have been characterized in in vivo studies. An overview of data obtained from in vivo preclinical trials, which addressed the use of these enzymes against animal models of human infections and diseases, is summarized in Table 3, organized by pathogen.

To date, there are currently no applications of phage-derived proteins approved or in Phase III in both European Union and USA [121]. However, several placebo-controlled clinical trials demonstrated safety of phage therapy, as the intravenous use of PlySs2 (CF-301) by ContraFect (ClinicalTrials.gov Identifier: NCT02439359), but no data have been published yet (Table 1).

The use of lysins as therapeutics offers distinct advantages compared to antibiotics for disease prevention and treatment. The biological action of these enzymes is rapid, as already described. They can be used on mucosae to kill colonizing pathogenic bacteria without altering the resident flora due to their specificity. For example, Nelson et al. described the activity of a lysin whose action is specific for streptococci of groups A, C, and E without any observed effect on several other oral streptococci or other commensal organisms of the upper respiratory tract [84]. Notably, they have less bacterial resistance issues than antibiotics [84] and seem to be effective as decontamination reagents [83]. Moreover, resistance development to endolysin catalytic activity is unlikely as there are no described cases of bacteria-losing sensitivity to these enzymes or resistant mutants obtained after in vitro exposure to sublethal concentrations of protein [122].

### 5.3. Virion-Associated Peptidoglycan Hydrolases (VAPGH)

Another phage-derived protein is represented by the virion-associated peptidoglycan hydrolase (VAPGH, or tail-associated lysin). These enzymes are structural components of some phage particles and mediate the local hydrolysis of cell wall peptidoglycan, allowing the phage tail tube to access the cytoplasm for viral DNA transfer [123, 124]. VAPGH are present in phages infecting both Gram-negative and Gram-positive bacteria but show a high degree of similarity to endolysins: these proteins also present a modular structure, allowing engineering to broaden and increase their bacteriolytic activity.

The chimeric enzyme P128 is the only VAPGH therapeutically tested in vivo. A MRSA strain was instilled into nares of rats followed by 2 intranasal treatments of a hydrogel formulation of P128 (50 mg/dose), or as a 2% mupirocin ointment (30 mg/dose). P128 hydrogel treatment was able to have MRSA colonization, and no efficacy was observed with the second formulation [125]. Later, GangaGen performed a combined Phase I and Phase II clinical trial evaluating the intranasal use of P128 (ClinicalTrials.gov Identifier NCT01746654), but no results have been published yet (Table 1).

### 5.4. Holins

As described, endolysins are responsible for peptidoglycan degradation at the final stages of cell lysis. Their activity involves another class of enzymes, named holins, which act in two different pathways: holin-endolysin and pinholin-SAR (signal anchor-release) endolysin systems. In the former system, these enzymes are phage-encoded proteins involved in the massive permeabilization of the cytoplasmic membrane, allowing endolysins to translocate into the periplasm and attack the peptidoglycan [81]. In the latter system, endolysins are exported into the periplasm and accumulate as inactive proteins. De-energization of the cytoplasmic membrane by imbedded pinholes activates SAR endolysins [81, 126] that are localised in the periplasm before pinholin triggering; thus, the degradation of peptidoglycan occurs more evenly, in contrast to the cell rupture in the first system [81].

The bacteriostatic activity of the holin-like protein Tmp1 was firstly demonstrated by Rajesh et al. [123]. This gene could complement a holin-defective phage *λ* and produce visible plaques on *E. coli* lawns. Its overexpression strongly inhibited *E. coli* growth during induction, and lysates inhibited the growth of different Gram-positive bacteria [123]. Surprisingly, holin-promoted lysis was observed in strains that were insensitive to endolysin. Thus, the holin-LySMP mixture was able to extend the spectrum of the endolysin alone, showing an interesting activity against several strains of multidrug-resistant *S. suis* and *S. aureus* [127].

## 6. Pathogen Detection

Because of the lack of strict regulation of their use as therapeutics, phage-based products are more widely considered for detection of pathogens in areas, such as food industry, water quality surveillance, or bioterrorism, where contaminations are frequently responsible of gastroenteritis, respiratory and mucosa infections, and if not worse, *sequelae.*

### 6.1. Bacteria Biosensors

Gastrointestinal epidemics, as the outbreak occurred in Germany in 2011 caused by Shiga toxin-producing strain *E. coli* O104:H4, revealed a critical need of efficient tools for pathogen detection. Standard microbiological methods for pathogen identification are time consuming; besides, molecular methods such as quantitative PCR (qPCR) or DNA hybridization require high-purity specimens [128]. Likewise, enzymatic assays such as ELISA are very sensitive but not suitable for high-throughput screenings. For these reasons, phage-based technology represents an alternative approach to prevent such outbreaks, and phages have been recently considered for biosensoring bacteria [129, 130]. To date, some systems have been developed with *E. coli* 0157:H7 as their target, and two main groups of applications are available: one uses inert phage particles or their proteins, the other requires infecting phages [131, 132]. In addition, phages are able to replicate at high titres and with low costs, they are more resistant to temperature or pH variations compared to antibodies and can be kept at room temperature without activity loss [133]. FASTPlaqueTB assay takes advantage of these characteristics for the detection of *Mycobacterium tuberculosis* in sputum: phages (Actiphages) that infect the slow-growing tubercle bacillus survive to the addiction of a virucide (Virusol) and are then detected through their plaques on nonpathogenic mycobacterial sensor cells [134].

### 6.2. Labelled Phages

Ulitzur and Kuhn (1989) were the first to incorporate a reporter for the bacterial luciferase gene (lux) into phage *λ* for detection of *E. coli*, allowing quick and sensitive detection by luminometer after its expression in bacterial culture. Other examples are the Listeria bacteriophage A511, in which Vibrio harveyi luxAB genes are inserted downstream of the major capsid protein, or bacteriophage for *Yersinia pestis* and *Bacillus anthracis*, whose rapid diagnosis is crucial [129, 130]. Luciferase assay is a fast and low-cost test, as it requires only 1 or 2 days to estimate luciferase expression into target cells detecting bioluminescence [135], so it can be used also in developing country laboratories [136]. Unfortunately, bacteriophage typing has important limitations such as phage resistance issues and difficulties related to phage stocks and propagating strains maintenance [137, 138]. In addition, other types of reporter genes can be inserted in phage genomes. For example, hyperthermophilic *β*-glycosidase gene has been tested for *Listeria* detection, with the important advantage that the amplification signal goes on as long as the substrate is provided, also when the host has already been lysed [139].

On the other hand, labelled phages can be chemically or genetically modified adding a fluorescent dye, nanoparticle, or protein, covalently bound onto the phage-coat surface in order to identify target bacteria by fluorescence microscopy. For example, Awais et al. constructed GFP-labelled phage PP01 specific to *E. coli* O157:H7: when propagated in bacteria, the intensity of green fluorescence increases. In addition, these phages have a defective lysozyme, so they are unable to mediate host lysis in order to avoid signal reduction due to bacteria lysis [131].

However, only a few phage kits for pathogen detection in human samples have reached the market, such as the previously described FASTPlaqueTB assay and the MRSA/MSSA Blood Culture Test (http://www.microphage.com/technology/) [140]. This is because these detection systems have to be disposable and single-use kits; thus, the nature of their components, especially if genetically modified, presents safety issues. Their usage must be constrained within an appropriate waste management protocol.

## 7. Conclusions

Bacteriophages, the most ubiquitous organisms on Earth, are viruses that infect bacteria and, for that reason, have been employed since their discovery in the development of therapeutics against infections. They are highly specific, very safe, and effective against their target pathogenic bacteria and rapidly modifiable in order to address new threats.

The advent of antibiotics, which saved patients' lives and had a pivotal role in the achievement of considerable advances in medicine and surgery, made this approach less exploited. Moreover, bacteriophage development has been obstructed by reduced financial resources, by scientific and regulatory hurdles (such as lack of quality control and of properly controlled studies), and by unfamiliarity with phages themselves. However, the emergence of an increasing number of antibiotic resistant species forced modern medicine to propose novel therapeutic strategies, and research on these viruses bloomed again.

Here, we presented an overview of different current applications of bacteriophages and their usages against infectious diseases. Phage display techniques allow the identification of neutralizing mAbs against several pathogens and have had a pivotal role in the rational design of effective vaccines. Phage therapy approaches, which take advantage of lytic phage characteristics, have inspired the start of many human clinical trials that have great potential as novel treatments of bacterial infections (Table 1). Phage-derived proteins gained appreciation as antibacterial agents and put their relevance into effect through polysaccharide depolymerases, endolysins, engineered endolysins, virion-associated peptidoglycan hydrolases, and holins. Finally, bacteriophages have proved useful as biosensors in pathogen detection.

On the other hand, because of their narrow specificity, the isolation of therapeutic phages could be technically demanding, and their successful use strongly depends on the ability to promptly identify the etiologic agent and to ensure in vitro the lytic efficacy of preparations against the appropriate bacterial strain. The selection of phage-resistant mutants could also be a possible issue of phage therapy, even if the mixture of different phage (i.e., “phage cocktails”) should help preventing it by increasing the range of targets of phage preparations. In any case, phage production, purification, and characterization should be performed taking into account the latest findings and the state-of-the-art biotechnology.

In conclusion, although much work remains to be done, bacteriophages and their applications seem to be a valid alternative to traditional approaches for the prevention and treatment of bacterial epidemics.

## Figures and Tables

**Table 1:** Bacteriophage and phage-derived proteins tested in clinical trials.

Drug	Condition	Phase	Results	Identifier
WPP-201 bacteriophage	Venous leg ulcers	I	This study found no safety concerns with the bacteriophage treatment [57]	NCT00663091
T4 phage cocktail	Diarrhea	I/II	Oral phages showed a safe gut transit in children but failed to achieve intestinal amplification and to improve diarrhea outcome, possibly due to insufficient phage coverage and too low *E. coli* pathogen titres requiring higher oral phage doses [58]	NCT00937274
*E. coli* phage cocktail	Wound infection	I/II ongoing	No results published yet	NCT02116010
Topical anti-*Staphylococcus* bacteriophage therapy	Diabetic foot staphylococcal infections	I/II ongoing	No results published yet	NCT02664740
Lysin CF-301	*S. aureus* bloodstream infections	I ongoing	No results published yet	NCT02439359
VAPGH P128	Nasal *S. aureus* colonization *S. aureus*-infected venous ulcer	I/II completed	No results published yet	NCT01746654

**Table 2:** Biofilm control by EPS depolymerase studies in vitro.

Target pathogen	Observations	Concerns	References
*Pseudomonas putida*	Biofilm clearance: significant variations between bacterial strains upon biofilm aging	Reduction of aged biofilm susceptibility to phage infection because of their thickness and phenotypic changes	[74]
*Klebsiella pneumoniae*	Greater biofilm clearance if cotreated with ciprofloxacin	Possible inhibition of depolymerase activity by ciprofloxacin	[75]
*Escherichia coli*	Depolymerase-producing phage construct capable of biofilm clearance	Results obtained for an engineered T7 strain	[76]
*Enterobacter cloace*	Biofilm clearance with depolymerase-producing phage; enhancement of chemical antibacterial penetration after phage-free depolymerase treatment	Combinations of three phages required for eradication of single-species biofilms; ineffective treatment of dual-species biofilms	[77]
*Pseudomonas aeruginosa*	Old biofilm clearance (20 days)	Bacteriophage migration facilitated by reduction of alginate viscosity	[78]
*Enterobacter agglomerans* and *Serratia marcescens*	Phage-resistant bacteria biofilm clearance with treatment of phage or phage-free depolymerase	Little differences in the chemical composition of EPS prevent the degradation of the polymer	[79]
*Enterobacter agglomerans*	Dual-species biofilm clearance with phage-free depolymerase	Specific depolymerase: no degradation of single biofilms of *Klebsiella pneumoniae*'s EPS	[80]

**Table 3:** Summary of phage-encoded endolysins tested in vivo.

Target pathogen	Endolysin	Animal model	References
*Acinetobacter baumannii*	PlyF307	Bacteraemia	[97]
*Bacillus anthracis*	PlyG PlyPH	Sepsis Peritonitis	[98, 99]
*Pseudomonas aeruginosa*	LoGT-008 (Artilysin)	Gut decolonization	[100]
*Staphylococcus aureus*	ClyS *λ*Sa2-E-lyso-SH3b LysK/CHAPk LysGH15 MV-L PhiSH2 Phi11 PlySs2 Ply187AN-KSH3b SAL-1 Twort WMY 80*α* 2638A	Nasal decolonization Bacteraemia Sepsis Mastitis Endophthalmitis	[101–111]
*Streptococcus agalactiae*	PlyGBS/PlyGBS90–1 PlySK1249	Vaginal decolonization Oropharynx decolonization Bacteraemia	[112–114]
*Streptococcus pneumonia*	Cpl-1 Cpl-771 PAL	Bacteraemia Sepsis Endocarditis Meningitis	[115–120]
*Streptococcus pyogenes*	PlyC (formerly C1) PlyPy PlySs2	Oral decolonization Bacteraemia	[84, 97, 106]

## References

[1] Wommack K. E., Hill R. T., Kessel M., Russek-Cohen E., Colwell R. R. (1992). Distribution of viruses in the Chesapeake Bay. *Applied and Environmental Microbiology*.

[2] D'Herelle F., Twort F. W., Bordet J., Gratia A. (1922). Discussion on the bacteriophage (bacteriolysin). *BMJ*.

[3] Twort F. (1925). The discovery of the ‘bacteriophage’. *The Lancet*.

[4] Duckworth D. H. (1976). Who discovered bacteriophage?. *Bacteriological Reviews*.

[5] Giguère S., Prescott J. F., Dowling P. M. (2013). *Antimicrobial Therapy in Veterinary Medicine*.

[6] Henry K. A., Arbabi-Ghahroudi M., Scott J. K. (2015). Beyond phage display: non-traditional applications of the filamentous bacteriophage as a vaccine carrier, therapeutic biologic, and bioconjugation scaffold. *Frontiers in Microbiology*.

[7] Hofschneider P. H., Mueller-Jensen K. (1963). Über infektiöse Substrukturen aus *E. coli*-Bakteriophagen. *Zeitschrift für Naturforschung Part B*.

[8] Marvin D. A., Hoffmann-Berling H. (1963). A fibrous DNA phage (fd) and a spherical RNA phage (fr) specific for male strains of E coli. *Zeitschrift für Naturforschung Part B*.

[9] Zinder N. D., Valentine R. C., Roger M., Stoeckenius W. (1963). F1, a rod-shaped male-specific bacteriophage that contains DNA. *Virology*.

[10] Salivar W. O., Tzagoloff H., Pratt D. (1964). Some physical-chemical and biological properties of the rod-shaped coliphage M13. *Virology*.

[11] Fauquet C. M., Fargette D. (2005). International Committee on Taxonomy of Viruses and the 3, 142 unassigned species. *Virology Journal*.

[12] Danner S., Belasco J. G. (2001). T7 phage display: a novel genetic selection system for cloning RNA-binding proteins from cDNA libraries. *Proceedings of the National Academy of Sciences of the United States of America*.

[13] Chanishvili N. (2012). Phage therapy—history from Twort and d’Herelle through Soviet experience to current approaches. *Advances in Virus Research*.

[14] Summers W. C. (2001). Bacteriophage therapy. *Annual Review of Microbiology*.

[15] Bruynoghe R., Maisin J. (1921). Essais de thérapeutique au moyen du bacteriophage. *CR Society of Biology*.

[16] Luria S. E., Delbrück M., Anderson T. F. (1943). Electron microscope studies of bacterial viruses. *Journal of Bacteriology*.

[17] Luria S. E., Delbrück M. (1943). Mutations of bacteria from virus sensitivity to virus resistance. *Genetics*.

[18] Ventola C. L. (2015). The antibiotic resistance crisis: part 1: causes and threats. *P t.*.

[19] Clark J. R., March J. B. (2004). Bacterial viruses as human vaccines?. *Expert Review of Vaccines*.

[20] Clementi N., Criscuolo E., Castelli M., Clementi M. (2012). Broad-range neutralizing anti-influenza A human monoclonal antibodies: new perspectives in therapy and prophylaxis. *The new Microbiologica*.

[21] Menéndez T., Santiago-Vispo N. F., Cruz-Leal Y. (2011). Identification and characterization of phage-displayed peptide mimetics of Neisseria meningitidis serogroup B capsular polysaccharide. *International Journal of Medical Microbiology*.

[22] Sautto G., Mancini N., Diotti R. A., Solforosi L., Clementi M., Burioni R. (2012). Anti-hepatitis C virus E2 (HCV/E2) glycoprotein monoclonal antibodies and neutralization interference. *Antiviral Research*.

[23] Mir H. M., Birerdinc A., Younossi Z. M. (2009). Monoclonal and polyclonal antibodies against the HCV envelope proteins. *Clinics in Liver Disease*.

[24] Diotti R. A., Mancini N., Clementi N. (2014). Cloning of the first human anti-JCPyV/VP1 neutralizing monoclonal antibody: epitope definition and implications in risk stratification of patients under natalizumab therapy. *Antiviral Research*.

[25] Williamson R. A., Burioni R., Sanna P. P., Partridge L. J., Barbas C. F., Burton D. R. (1993). Human monoclonal antibodies against a plethora of viral pathogens from single combinatorial libraries. *Proceedings of the National Academy of Sciences of the United States of America*.

[26] Thompson J., Pope T., Tung J. S. (1996). Affinity maturation of a high-affinity human monoclonal antibody against the third hypervariable loop of human immunodeficiency virus: use of phage display to improve affinity and broaden strain reactivity. *Journal of Molecular Biology*.

[27] Cao J., Sun Y., Berglindh T. (2000). Helicobacter pylori-antigen-binding fragments expressed on the filamentous M13 phage prevent bacterial growth. *Biochimica et Biophysica Acta*.

[28] Burioni R., Williamson R. A., Sanna P. P., Bloom F. E., Burton D. R. (1994). Recombinant human Fab to glycoprotein D neutralizes infectivity and prevents cell-to-cell transmission of herpes simplex viruses 1 and 2 in vitro. *Proceedings of the National Academy of Sciences of the United States of America*.

[29] Lee C. C., Lin L. L., Chan W. E., Ko T.-P., Lai J. S., Wang A. H. J. (2013). Structural basis for the antibody neutralization of Herpes simplex virus. *Acta Crystallographica. Section D, Biological Crystallography*.

[30] Lindquist E. A., Marks J. D., Kleba B. J., Stephens R. S. (2002). Phage-display antibody detection of Chlamydia trachomatis-associated antigens. *Microbiology*.

[31] Paoli G. C., Chen C.Y., Brewster J. D. (2004). Single-chain Fv antibody with specificity for *Listeria monocytogenes*. *Journal of Immunological Methods*.

[32] Zhu Y., Ho B., Ding J. L. (2003). Sequence and structural diversity in endotoxin-binding dodecapeptides. *Biochimica et Biophysica Acta*.

[33] Zhao X.L., Yin J., Chen W.Q., Jiang M., Yang G., Yang Z.H. (2008). Generation and characterization of human monoclonal antibodies to G5, a linear neutralization epitope on glycoprotein of rabies virus, by phage display technology. *Microbiology and Immunology*.

[34] Sabarth N., Hurvitz R., Schmidt M. (2005). Identification of helicobacter pylori surface proteins by selective proteinase K digestion and antibody phage display. *Journal of Microbiological Methods*.

[35] Wu H., Pfarr D. S., Johnson S. (2007). Development of motavizumab, an ultra-potent antibody for the prevention of respiratory syncytial virus infection in the upper and lower respiratory tract. *Journal of Molecular Biology*.

[36] Ran S., He J., Huang X., Soares M., Scothorn D., Thorpe P. E. (2005). Antitumor effects of a monoclonal antibody that binds anionic phospholipids on the surface of tumor blood vessels in mice. *Clinical Cancer Research*.

[37] Migone T.-S., Subramanian G. M., Zhong J. (2009). Raxibacumab for the treatment of inhalational anthrax. *The New England Journal of Medicine*.

[38] Odegrip R., Coomber D., Eldridge B. (2004). CIS display: in vitro selection of peptides from libraries of protein-DNA complexes. *Proceedings of the National Academy of Sciences of the United States of America*.

[39] Steel J. (2011). New strategies for the development of H5N1 subtype influenza vaccines: progress and challenges. *BioDrugs*.

[40] Johnston C., Koelle D. M., Wald A. (2011). HSV-2: in pursuit of a vaccine. *The Journal of Clinical Investigation*.

[41] Danko J. R., Beckett C. G., Porter K. R. (2011). Development of dengue DNA vaccines. *Vaccine*.

[42] Klasse P. J., Sattentau Q. J. (2002). Occupancy and mechanism in antibody-mediated neutralization of animal viruses. *The Journal of General Virology*.

[43] Dulbecco R., Vogt M., Strickland A. G. (1956). A study of the basic aspects of neutralization of two animal viruses, western equine encephalitis virus and poliomyelitis virus. *Virology*.

[44] Takada A., Kawaoka Y. (2003). Antibody-dependent enhancement of viral infection: molecular mechanisms and in vivo implications. *Reviews in Medical Virology*.

[45] O’Rourke J. P., Peabody D. S., Chackerian B. (2015). Affinity selection of epitope-based vaccines using a bacteriophage virus-like particle platform. *Current Opinion in Virology*.

[46] Castelli M., Cappelletti F., Diotti R. A. (2013). Peptide-based vaccinology: experimental and computational approaches to target hypervariable viruses through the fine characterization of protective epitopes recognized by monoclonal antibodies and the identification of T-cell-activating peptides. *Clinical and Developmental Immunology*.

[47] Vukovic P., Chen K., Qin Liu X. (2002). Single-chain antibodies produced by phage display against the C-terminal 19 kDa region of merozoite surface protein-1 of *Plasmodium yoelii* reduce parasite growth following challenge. *Vaccine*.

[48] Bugli F., Mancini N., Kang C. Y. (2001). Mapping B-cell epitopes of hepatitis C virus E2 glycoprotein using human monoclonal antibodies from phage display libraries. *Journal of Virology*.

[49] Hashemi H., Bamdad T., Jamali A., Pouyanfard S., Mohammadi M. G. (2010). Evaluation of humoral and cellular immune responses against HSV-1 using genetic immunization by filamentous phage particles: a comparative approach to conventional DNA vaccine. *Journal of Virological Methods*.

[50] Aghebati-Maleki L., Bakhshinejad B., Baradaran B. (2016). Phage display as a promising approach for vaccine development. *Journal of Biomedical Science*.

[51] Tao P., Mahalingam M., Kirtley M. L. (2013). Mutated and bacteriophage T4 nanoparticle arrayed F1-V immunogens from Yersinia pestis as next generation plague vaccines. *PLoS Pathogens*.

[52] Sathaliyawala T., Rao M., Maclean D. M., Birx D. L., Alving C. R., Rao V. B. (2006). Assembly of human immunodeficiency virus (HIV) antigens on bacteriophage T4: a novel in vitro approach to construct multicomponent HIV vaccines. *Journal of Virology*.

[53] Hashemi H., Pouyanfard S., Bandehpour M. (2012). Immunization with M2e-displaying T7 bacteriophage nanoparticles protects against influenza A virus challenge. *PloS One*.

[54] Clark J. R., March J. B. (2004). Bacteriophage-mediated nucleic acid immunisation. *FEMS Immunology and Medical Microbiology*.

[55] Ou C., Tian D., Ling Y. (2013). Evaluation of an ompA-based phage-mediated DNA vaccine against Chlamydia abortus in piglets. *International Immunopharmacology*.

[56] Latz S., Wahida A., Arif A. (2016). Preliminary survey of local bacteriophages with lytic activity against multi-drug resistant bacteria. *Journal of Basic Microbiology*.

[57] Rhoads D. D., Wolcott R. D., Kuskowski M. A., Wolcott B. M., Ward L. S., Sulakvelidze A. (2013). Bacteriophage therapy of venous leg ulcers in humans: results of a phase I safety trial. *Journal of Wound Care*.

[58] Sarker S. A., Sultana S., Reuteler G. (2016). Oral phage therapy of acute bacterial diarrhea with two coliphage preparations: a randomized trial in children from Bangladesh. *eBioMedicine*.

[59] Oliveira H., Sillankorva S., Merabishvili M., Kluskens L. D., Azeredo J. (2015). Unexploited opportunities for phage therapy. *Frontiers in Pharmacology*.

[60] Nilsson A. S. (2014). Phage therapy—constraints and possibilities. *Upsala Journal of Medical Sciences*.

[61] Hodyra-Stefaniak K., Miernikiewicz P., Drapała J. (2015). Mammalian host-versus-phage immune response determines phage fate in vivo. *Scientific Reports*.

[62] Ryan E. M., Gorman S. P., Donnelly R. F., Gilmore B. F. (2011). Recent advances in bacteriophage therapy: how delivery routes, formulation, concentration and timing influence the success of phage therapy. *The Journal of Pharmacy and Pharmacology*.

[63] Drulis-Kawa Z., Majkowska-Skrobek G., Maciejewska B. (2015). Bacteriophages and phage-derived proteins—application approaches. *Current Medicinal Chemistry*.

[64] Flemming H.-C., Wingender J., Szewzyk U., Steinberg P., Rice S. A., Kjelleberg S. (2016). Biofilms: an emergent form of bacterial life. *Nature Reviews. Microbiology*.

[65] Harada T. (1983). Special bacterial polysaccharides and polysaccharases. *Biochemical Society Symposium*.

[66] Dubos R., Avery O. T. (1931). Decomposition of the capsular polysaccharide of pneumococcus type III by a bacterial enzyme. *The Journal of Experimental Medicine*.

[67] Goodner K., Dubos R. (1932). Studies on the quantitative action of a specific enzyme in type III pneumococcus dermal infection in rabbits. *The Journal of Experimental Medicine*.

[68] Francis T., Terrell E. E., Dubos R., Avery O. T. (1934). Experimental type III pneumococcus pneumonia in monkeys: II. Treatment with an enzyme which decomposes the specific capsular polysaccharide of pneumococcus type III. *The Journal of Experimental Medicine*.

[69] Mushtaq N., Redpath M. B., Luzio J. P., Taylor P. W. (2005). Treatment of experimental *Escherichia coli* infection with recombinant bacteriophage-derived capsule depolymerase. *The Journal of Antimicrobial Chemotherapy*.

[70] Scorpio A., Chabot D. J., Day W. A. (2007). Poly-gamma-glutamate capsule-degrading enzyme treatment enhances phagocytosis and killing of encapsulated bacillus anthracis. *Antimicrobial Agents and Chemotherapy*.

[71] Scorpio A., Tobery S. A., Ribot W. J., Friedlander A. M. (2008). Treatment of experimental anthrax with recombinant capsule depolymerase. *Antimicrobial Agents and Chemotherapy*.

[72] Zelmer A., Martin M. J., Gundogdu O. (2010). Administration of capsule-selective endosialidase E minimizes upregulation of organ gene expression induced by experimental systemic infection with *Escherichia coli* K1. *Microbiology*.

[73] Bales P. M., Renke E. M., May S. L., Shen Y., Nelson D. C. (2013). Purification and characterization of biofilm-associated EPS exopolysaccharides from ESKAPE organisms and other pathogens. *PloS One*.

[74] Cornelissen A., Ceyssens P.-J., T'Syen J. (2011). The T7-related Pseudomonas putida phage φ15 displays virion-associated biofilm degradation properties. *PloS One*.

[75] Verma V., Harjai K., Chhibber S. (2010). Structural changes induced by a lytic bacteriophage make ciprofloxacin effective against older biofilm of *Klebsiella pneumoniae*. *Biofouling*.

[76] Lu T. K., Collins J. J. (2007). Dispersing biofilms with engineered enzymatic bacteriophage. *Proceedings of the National Academy of Sciences of the United States of America*.

[77] Tait K., Skillman L. C., Sutherland I. W. (2010). The efficacy of bacteriophage as a method of biofilm eradication. *Biofouling*.

[78] Hanlon G. W., Denyer S. P., Olliff C. J., Ibrahim L. J. (2001). Reduction in exopolysaccharide viscosity as an aid to bacteriophage penetration through Pseudomonas aeruginosa biofilms. *Applied and Environmental Microbiology*.

[79] Hughes K. A., Sutherland I. W., Jones M. V. (1998). Biofilm susceptibility to bacteriophage attack: the role of phage-borne polysaccharide depolymerase. *Microbiology*.

[80] Skillman L. C., Sutherland I. W., Jones M. V. (1998). The role of exopolysaccharides in dual species biofilm development. *Journal of Applied Microbiology*.

[81] Young R. (2014). Phage lysis: three steps, three choices, one outcome. *Journal of Microbiology*.

[82] Zhang L., Li D., Li X. (2016). LysGH15 kills Staphylococcus aureus without being affected by the humoral immune response or inducing inflammation. *Scientific Reports*.

[83] Fischetti V. A. (2005). Bacteriophage lytic enzymes: novel anti-infectives. *Trends in Microbiology*.

[84] Nelson D., Loomis L., Fischetti V. A. (2001). Prevention and elimination of upper respiratory colonization of mice by group A streptococci by using a bacteriophage lytic enzyme. *Proceedings of the National Academy of Sciences of the United States of America*.

[85] Briers Y., Lavigne R. (2015). Breaking barriers: expansion of the use of endolysins as novel antibacterials against Gram-negative bacteria. *Future Microbiology*.

[86] Yuan Y., Peng Q., Gao M. (2012). Characteristics of a broad lytic spectrum endolysin from phage BtCS33 of Bacillus thuringiensis. *BMC Microbiology*.

[87] Roach D. R., Donovan D. M. (2015). Antimicrobial bacteriophage-derived proteins and therapeutic applications. *Bacteriophage.*.

[88] Schmelcher M., Donovan D. M., Loessner M. J. (2012). Bacteriophage endolysins as novel antimicrobials. *Future Microbiology*.

[89] Loessner M. J., Kramer K., Ebel F., Scherer S. (2002). C-terminal domains of *Listeria monocytogenes* bacteriophage murein hydrolases determine specific recognition and high-affinity binding to bacterial cell wall carbohydrates. *Molecular Microbiology*.

[90] Callewaert L., Walmagh M., Michiels C. W., Lavigne R. (2011). Food applications of bacterial cell wall hydrolases. *Current Opinion in Biotechnology*.

[91] Merzlyak A. (2009). *Development and Characterization of Genetically Engineered M13 Bacteriophage as Tissue Engineering Materials*.

[92] Walmagh M., Boczkowska B., Grymonprez B., Briers Y., Drulis-Kawa Z., Lavigne R. (2013). Characterization of five novel endolysins from gram-negative infecting bacteriophages. *Applied Microbiology and Biotechnology*.

[93] Lukacik P., Barnard T. J., Buchanan S. K. (2012). Using a bacteriocin structure to engineer a phage lysin that targets Yersinia pestis. *Biochemical Society Transactions*.

[94] Briers Y., Walmagh M., Grymonprez B. (2014). Art-175 is a highly efficient antibacterial against multidrug-resistant strains and persisters of Pseudomonas aeruginosa. *Antimicrobial Agents and Chemotherapy*.

[95] Walmagh M., Briers Y., Santos, dos, S.B., Azeredo, J., Lavigne, R. (2012). Characterization of modular bacteriophage endolysins from Myoviridae phages OBP, 201φ2-1 and PVP-SE1. *PloS One*.

[96] Freimer E. H., Krause R. M., McCarty M. (1959). Studies of L forms and protoplasts of group A streptococci. I. Isolation, growth, and bacteriologic characteristics. *The Journal of Experimental Medicine*.

[97] Lood R., Winer B. Y., Pelzek A. J. (2015). Novel phage lysin capable of killing the multidrug-resistant gram-negative bacterium Acinetobacter baumannii in a mouse bacteremia model. *Antimicrobial Agents and Chemotherapy*.

[98] Schuch R., Nelson D., Fischetti V. A. (2002). A bacteriolytic agent that detects and kills bacillus anthracis. *Nature*.

[99] Yoong P., Schuch R., Nelson D., Fischetti V. A. (2006). PlyPH, a bacteriolytic enzyme with a broad pH range of activity and lytic action against Bacillus anthracis. *Journal of Bacteriology*.

[100] Briers Y., Walmagh M., Van Puyenbroeck V. (2014). Engineered endolysin-based “Artilysins” to combat multidrug-resistant gram-negative pathogens. *MBio*.

[101] Rashel M., Uchiyama J., Ujihara T. (2007). Efficient elimination of multidrug-resistant *Staphylococcus aureus* by cloned lysin derived from bacteriophage phi MR11. *The Journal of Infectious Diseases*.

[102] Daniel A., Euler C., Collin M., Chahales P., Gorelick K. J., Fischetti V. A. (2010). Synergism between a novel chimeric lysin and oxacillin protects against infection by methicillin-resistant *Staphylococcus aureus*. *Antimicrobial Agents and Chemotherapy*.

[103] Pastagia M., Euler C., Chahales P., Fuentes-Duculan J., Krueger J. G., Fischetti V. A. (2011). A novel chimeric lysin shows superiority to mupirocin for skin decolonization of methicillin-resistant and -sensitive *Staphylococcus aureus* strains. *Antimicrobial Agents and Chemotherapy*.

[104] Gu J., Xu W., Lei L. (2011). LysGH15, a novel bacteriophage lysin, protects a murine bacteremia model efficiently against lethal methicillin-resistant *Staphylococcus aureus* infection. *Journal of Clinical Microbiology*.

[105] Gu J., Zuo J., Lei L. (2011). LysGH15 reduces the inflammation caused by lethal methicillin-resistant *Staphylococcus aureus* infection in mice. *Bioengineered Bugs*.

[106] Gilmer D. B., Schmitz J. E., Euler C. W., Fischetti V. A. (2013). Novel bacteriophage lysin with broad lytic activity protects against mixed infection by Streptococcus pyogenes and methicillin-resistant *Staphylococcus aureus*. *Antimicrobial Agents and Chemotherapy*.

[107] Schmelcher M., Powell A. M., Becker S. C., Camp M. J., Donovan D. M. (2012). Chimeric phage lysins act synergistically with lysostaphin to kill mastitis-causing *Staphylococcus aureus* in murine mammary glands. *Applied and Environmental Microbiology*.

[108] Schmelcher M., Shen Y., Nelson D. C. (2015). Evolutionarily distinct bacteriophage endolysins featuring conserved peptidoglycan cleavage sites protect mice from MRSA infection. *The Journal of Antimicrobial Chemotherapy*.

[109] Singh P. K., Donovan D. M., Kumar A. (2014). Intravitreal injection of the chimeric phage endolysin Ply187 protects mice from *Staphylococcus aureus* endophthalmitis. *Antimicrobial Agents and Chemotherapy*.

[110] Jun S. Y., Jung G. M., Yoon S. J. (2013). Antibacterial properties of a pre-formulated recombinant phage endolysin, SAL-1. *International Journal of Antimicrobial Agents*.

[111] Jun S. Y., Jung G. M., Yoon S. J. (2014). Preclinical safety evaluation of intravenously administered SAL200 containing the recombinant phage endolysin SAL-1 as a pharmaceutical ingredient. *Antimicrobial Agents and Chemotherapy*.

[112] Cheng Q., Fischetti V. A. (2007). Mutagenesis of a bacteriophage lytic enzyme PlyGBS significantly increases its antibacterial activity against group B streptococci. *Applied Microbiology and Biotechnology*.

[113] Cheng Q., Nelson D., Zhu S., Fischetti V. A. (2005). Removal of group B streptococci colonizing the vagina and oropharynx of mice with a bacteriophage lytic enzyme. *Antimicrobial Agents and Chemotherapy*.

[114] Oechslin F., Daraspe J., Giddey M., Moreillon P., Resch G. (2013). In vitro characterization of PlySK1249, a novel phage lysin, and assessment of its antibacterial activity in a mouse model of *Streptococcus agalactiae* bacteremia. *Antimicrobial Agents and Chemotherapy*.

[115] Entenza J. M., Loeffler J. M., Grandgirard D., Fischetti V. A., Moreillon P. (2005). Therapeutic effects of bacteriophage Cpl-1 lysin against Streptococcus pneumoniae endocarditis in rats. *Antimicrobial Agents and Chemotherapy*.

[116] Pérez-Dorado I., Campillo N. E., Monterroso B. (2007). Elucidation of the molecular recognition of bacterial cell wall by modular pneumococcal phage endolysin CPL-1. *The Journal of Biological Chemistry*.

[117] Diez-Martinez R., De Paz H. D., García-Fernández E. (2015). A novel chimeric phage lysin with high in vitro and in vivo bactericidal activity against Streptococcus pneumoniae. *The Journal of Antimicrobial Chemotherapy*.

[118] Diez-Martinez R., De Paz H. D., de Paz H. (2013). Improving the lethal effect of cpl-7, a pneumococcal phage lysozyme with broad bactericidal activity, by inverting the net charge of its cell wall-binding module. *Antimicrobial Agents and Chemotherapy*.

[119] McCullers J. A., Karlström A., Iverson A. R., Loeffler J. M., Fischetti V. A. (2007). Novel strategy to prevent otitis media caused by colonizing Streptococcus pneumoniae. *PLoS Pathogens*.

[120] Loeffler J. M., Fischetti V. A. (2003). Synergistic lethal effect of a combination of phage lytic enzymes with different activities on penicillin-sensitive and -resistant Streptococcus pneumoniae strains. *Antimicrobial Agents and Chemotherapy*.

[121] Cooper C. J., Khan Mirzaei M., Nilsson A. S. (2016). Adapting drug approval pathways for bacteriophage-based therapeutics. *Frontiers in Microbiology*.

[122] Nakonieczna A., Cooper C. J., Gryko R. (2015). Bacteriophages and bacteriophage-derived endolysins as potential therapeutics to combat gram-positive spore forming bacteria. *Journal of Applied Microbiology*.

[123] Rajesh T., Anthony T., Saranya S., Pushpam P. L., Gunasekaran P. (2011). Functional characterization of a new holin-like antibacterial protein coding gene tmp1 from goat skin surface metagenome. *Applied Microbiology and Biotechnology*.

[124] Kenny J. G., McGrath S., Fitzgerald G. F., van Sinderen D. (2004). Bacteriophage Tuc2009 encodes a tail-associated cell wall-degrading activity. *Journal of Bacteriology*.

[125] Vipra A. A., Desai S. N., Roy P. (2012). Antistaphylococcal activity of bacteriophage derived chimeric protein P128. *BMC Microbiology*.

[126] Pang T., Fleming T. C., Pogliano K., Young R. (2013). Visualization of pinholin lesions in vivo. *Proceedings of the National Academy of Sciences*.

[127] Shi Y., Li N., Yan Y. (2012). Combined antibacterial activity of phage lytic proteins holin and lysin from Streptococcus suis bacteriophage SMP. *Current Microbiology*.

[128] Olson N. D., Morrow J. B. (2012). DNA extract characterization process for microbial detection methods development and validation. *BMC Research Notes*.

[129] Loessner M. J., Rees C. E., Stewart G. S., Scherer S. (1996). Construction of luciferase reporter bacteriophage A511:luxAB for rapid and sensitive detection of viable Listeria cells. *Applied and Environmental Microbiology*.

[130] Schofield D. A., Molineux I. J., Westwater C. (2011). “Bioluminescent” reporter phage for the detection of category a bacterial pathogens. *Journal of Visualized Experiments*.

[131] Awais R., Fukudomi H., Miyanaga K., Unno H., Tanji Y. (2006). A recombinant bacteriophage-based assay for the discriminative detection of culturable and viable but nonculturable *Escherichia coli* O157:H7. *Biotechnology Progress*.

[132] Anany H., Chen W., Pelton R., Griffiths M. W. (2011). Biocontrol of *Listeria monocytogenes* and *Escherichia coli* O157:H7 in meat by using phages immobilized on modified cellulose membranes. *Applied and Environmental Microbiology*.

[133] Bardy P., Pantůček R., Benešík M., Doškař J. (2016). Genetically modified bacteriophages in applied microbiology. *Journal of Applied Microbiology*.

[134] Albert H., Heydenrych A., Mole R., Trollip A., Blumberg L. (2001). Evaluation of FASTPlaqueTB-RIF, a rapid, manual test for the determination of rifampicin resistance from Mycobacterium tuberculosis cultures. *The International Journal of Tuberculosis and Lung Disease*.

[135] Smietana M., Bock W. J., Mikulic P., Ng A., Chinnappan R., Zourob M. (2011). Detection of bacteria using bacteriophages as recognition elements immobilized on long-period fiber gratings. *Optics Express*.

[136] Traore H., Ogwang S., Mallard K. (2007). Low-cost rapid detection of rifampicin resistant tuberculosis using bacteriophage in Kampala, Uganda. *Annals of Clinical Microbiology and Antimicrobials*.

[137] Tenover F. C., Arbeit R., Archer G. (1994). Comparison of traditional and molecular methods of typing isolates of *Staphylococcus aureus*. *Journal of Clinical Microbiology*.

[138] van Belkum A., Dunne W. M. (2013). Next-generation antimicrobial susceptibility testing. *Journal of Clinical Microbiology*.

[139] Hagens S., de Wouters T., Vollenweider P., Loessner M. J. (2011). Reporter bacteriophage A511:celB transduces a hyperthermostable glycosidase from Pyrococcus furiosus for rapid and simple detection of viable Listeria cells. *Bacteriophage*.

[140] Sullivan K. V., Turner N. N., Roundtree S. S., McGowan K. L. (2013). Rapid detection of methicillin-resistant *Staphylococcus aureus* (MRSA) and methicillin-susceptible *Staphylococcus aureus* (MSSA) using the KeyPath MRSA/MSSA blood culture test and the BacT/ALERT system in a pediatric population. *Archives of Pathology & Laboratory Medicine*.

